# Do dentists practice what they know? A cross-sectional study on the agreement between dentists' knowledge and practice in restoring endodontically treated teeth

**DOI:** 10.1186/s12903-021-01479-2

**Published:** 2021-03-10

**Authors:** Rua S. Babaier, Sumaya O. Basudan

**Affiliations:** 1 Department of Prosthetic Dental Sciences, College of Dentistry, King Saud University, Riyadh, 12372 Saudi Arabia; 2 Department of Restorative Dental Sciences, College of Dentistry, King Saud University, Riyadh, Saudi Arabia; 3 Present Address: Division of Dentistry, Faculty of Biology, Medicine, and Health, School of Medical Sciences, University of Manchester, Manchester, UK

**Keywords:** Patient care, Practice gap, Evidence-based dentistry, Post and core, Root canal therapy, Post space

## Abstract

**Background:**

There are very few studies comparing dentists' knowledge in relation to their clinical approach despite the existence of a possible gap between what they know and what they do.

**Aim:**

To measure the agreement between knowledge and practice methods related to a selected clinical scenario involving the placement of an indirect post in endodontically treated teeth (ETT) among different types of practitioners.

**Methods:**

An electronic questionnaire was emailed to members of the Saudi Dental Society. The questionnaire presented a clinical scenario of restoring a posterior ETT with an indirect post, core unit, and crown, followed by specific questions regarding knowledge and practice related to ten different treatment aspects such as who prepares the post space, technique, isolation, time, gap between gutta-percha, and time to cementation of the crown. Each question was presented twice for each aspect, once asking about their practice method and then what they thought was the correct practice (knowledge). The relationship between the participants' responses and their specialty and the agreement between the responses of knowledge and practice for each participant were analyzed by Pearson's chi-square test and Kappa.

**Results:**

203 completed questionnaires were analyzed. Most participants were 30 years old or younger (62.6%), and general dental practitioners (59%). When comparing the knowledge to the practice methods of each participant, nine out of ten aspects were of a "weak" level agreement or below (kappa < 0.59, *p* < 0.001). Only one aspect demonstrated a "strong" level of agreement (Kappa = 0.804), which was related to the duration of time between obturation and post space preparation in the presence of a periapical lesion. However, this strong agreement in the responses was not aligned with current evidence. There was also a significant difference among the responses of endodontists, restorative dentists and general practitioners in most of the aspects.

**Conclusion:**

Overall, there was a weak agreement between what practitioners know and do in most aspects of a selected clinical scenario involving the placement of an indirect post in posterior ETT. Moreover, the participant's specialty influenced their responses regarding both knowledge and clinical practice.

**Supplementary Information:**

The online version contains supplementary material available at 10.1186/s12903-021-01479-2.

## Background

The restorative treatment options for posterior teeth that undergo root canal treatment (RCT) vary in complexity from a minimal filling to an indirect post and crown. The reported success rate of endodontically treated teeth (ETT) is high; however, the treatment outcome depends on the quality of not only the endodontic treatment but also the coronal restoration. Therefore, it has become important to investigate the different treatment philosophies and practices related to this topic among dentists with various backgrounds [1–5].

Survey-based studies are a valuable tool for identifying the levels of knowledge, attitudes, and practices of clinicians, highlighting the adherence of practitioners to current recommendations or best practices. Earlier surveys evaluated prosthodontic-related aspects among clinicians such as the need for cuspal coverage, ferrule effect, the rationale for post-placement and technique for post space preparation (PSP), remaining apical seal, and types of posts, cement and core materials [6–13]. While RCT and subsequent restorative treatment can be performed by a general dental practitioner (GDP) or a more specialized practitioner such as an endodontist or a restorative specialist, an overlap exists between the different disciplines during PSP, since any of the groups can prepare it. Their expertise or specialization may influence the approaches and treatment decisions of practitioners. Leakage, for example, especially coronally, is detrimental to the success of ETT and maybe a major concern during different steps of treatment [14]. However, there is limited knowledge of different practitioners' perspectives and approaches towards the multiple aspects of PSP.

Surveys on the restorative management of ETT have investigated clinicians' knowledge of certain treatment aspects or their clinical practices [6–13]. However, both knowledge and practice have not been studied simultaneously or compared. Although knowledge and practice should ideally be in agreement, the literature has demonstrated that in health care, a gap between knowledge "know-what" and practice "do" may exist [15]. Most commonly, this gap has been explored through the "Knowledge Translation" model which is described as "a dynamic and iterative process that includes the synthesis, dissemination, exchange and ethically-sound application of knowledge to improve the health of Canadians, provide more effective health services and products and strengthen the healthcare system" [16, 17]. Accordingly, discrepancies in knowledge and practice can be explained by the difficulties practitioners face in knowledge application. We investigate here if such a gap does indeed exist in the management of ETT. To the best of our knowledge, there are no studies that have compared dentists' knowledge to their clinical approach.

Therefore, the aim of this study is twofold:

1. To measure the agreement between knowledge and practice methods related to a selected clinical scenario involving indirect post-placement in posterior ETT.
2. To compare the knowledge and practice responses between the different types of practitioners.

## Methods

The research protocol was in accordance with the principles of the World Medical Association Declaration of Helsinki and was approved by the Institutional Review Board and the College of Dentistry Research Center. A self-administered web-based questionnaire was created on a survey website www.FreeOnlineSurveys.com and emailed to the Saudi Dental Society (SDS). This official scientific society includes around 2500 active members from the dental field, such as dentists, dental assistants, technicians, hygienists, and dental students [18]. To reach out for our target participants, a covering letter invited dental practitioners involved in ETT management, including GDPs, endodontists, and restorative specialists such as prosthodontists and operative specialists. The objective of the survey and contact information of the researchers were included in the informed consent. Anonymity and confidentiality were assured, along with the voluntary nature of study participation. Responses were collected over two months, with an email reminder sent three weeks after the first request. There was no accurate data on the number of each specialty category within the SDS system. Therefore, the sample size was estimated based on the number of all active SDS members to be 250 with a confidence level of 95% and 5% error margin.

The questionnaire draft was derived from published studies and guided by experts' opinions (GDPs, Restorative specialists, and Endodontists) [7, 8, 10, 11]. The experts and skilled biostatistician evaluated face and content validity. The questionnaire was then piloted and adjusted for clarity. Reliability was calculated to be 0.75 (See Additional file 1: Supplementary file). The questionnaire was composed of the following three parts: part one was related to the demographic information of the participants; part two was related to the need and frequency of the placement of coronal cuspal restorations on ETT, which was answered on a sliding bar from 0 to 100; and part three addressed specific issues related to a clinical scenario for restoring posterior ETT in which an indirectly fabricated post, core unit, and crown were indicated without a need for a crown lengthening procedure. The responses to questions on the frequency of post and crown placement in posterior ETT were categorized as follows: rarely: 0–20%, sometimes: 30–50%, and more frequently: 60–100%. The clinical case presented a selected restorative treatment with a specific approach to standardize different possible variables aiming to examine the participants' philosophies more closely on a case with limited options regardless of other best treatment options. Multiple-choice questions were related to the time and method of preparation, remaining gutta-percha, presence of periapical lesions, practitioner preparing the post space, and presence of gaps, isolation, and temporization.

Each aspect was presented as two questions: the first question addressed how the participant manages a specific clinical situation (practice). The second was what the participant thought was the best way to handle the same situation (knowledge). For example, the questions for aspect #2 in the questionnaire were as follows:

- Do you routinely apply a rubber dam during post space preparation?
- Do you think it is recommended to place a rubber dam during post space preparation routinely?

### Data analysis

The data were analyzed using Statistical Package for Social Sciences software, version 21.0 (IBM Corp., New York, USA). Descriptive statistics (frequencies and percentages) were used to describe the categorical variables. Pearson's chi-square test was used to analyze the relationship of the participants' responses to their specialty, years of experience, and workplace. Kappa statistics were computed to observe the agreement between the responses of knowledge and practice aspects for each participant. Strength of agreement was interpreted following McHugh [19]: 0–0.20, none; 0.21–0.39, minimal; 0.40–0.59, weak; 0.60–0.79, moderate; 0.80–0.90, strong; and above 0.90, almost perfect. Statistical significance was set at 0.05 (*p* ≤ 0.05).

## Results

### Demographics

**Fig. 1:**
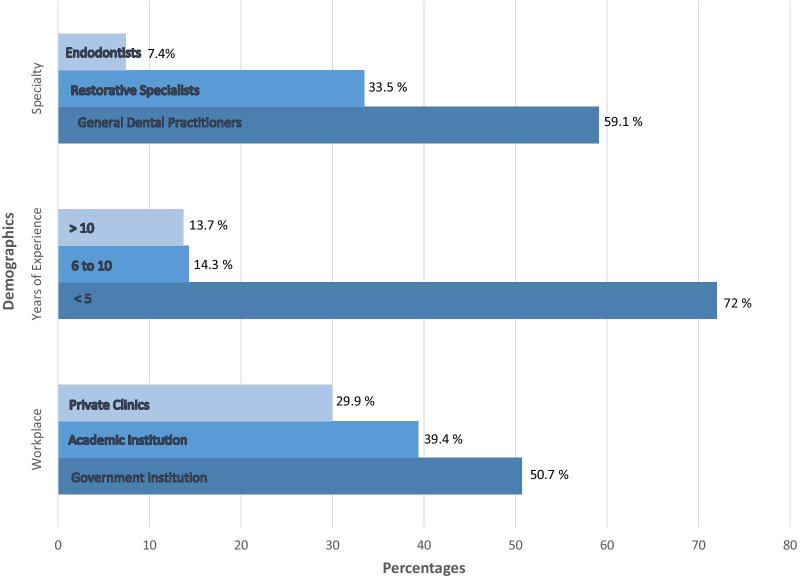
Distribution of sociodemographic characteristics of a sample of Saudi dentists (n = 203)

Of the returned questionnaires, 44 were excluded either because they were incomplete or because the participants were not involved in the restoration of ETT (e.g., dental hygienists and orthodontists), which resulted in 203 completed questionnaires. The response rate could not be calculated since the original number of SDS members eligible to participate unknown. Of the 203 participants, 127 (62.6%) were aged 30 years or younger, 56 (27.6%) were between 31 and 40 years, and 20 (9.9%) were older than 40 years. There were more females (67%) and participants of Saudi nationality (85.2%) than any other group. There were more GDPs (n = 120), than Restorative Specialists (n = 68) or Endodontists (n = 15). Figure 1 presents the distribution of participants according to specialty, years of experience and working sector.

### General restorative management of posterior ETT

There was a general tendency towards providing cuspal protection to teeth with root canal treatment among the different participants. However, specialists placed crowns more frequently on posterior ETT than GDPs (Table 1).

**Table 1:** Association between the responses towards the general restorative management of the posterior ETT and the specialty of a sample of Saudi dentists (n = 203)

General restorative management of posterior ETT	Specialty n (%)		
	GDP	Resto	Endo
*Frequency of crowning*			
Rarely	7(5.8)	1(1.5)	0
Sometimes	31(25.8)	6(8.8)	2(13.3)
More frequently	82(68.3)	61(89.7)	13(86.7)
*The need for cuspal coverage on all posterior ETT*			
Rarely	8(6.6)	6(8.8)	1(6.7)
Sometimes	39(32.5)	4(5.9)	1(6.7)
More frequently	73(60.8)	58(85.3)	13(86.7)
*Frequency of post-placement*			
Rarely	33(27.5)	7(10.3)	8(53.3)
Sometimes	44(36.7)	21(30.9)	4(26.7)
More frequently	43(35.8)	40(58.8)	3(20.0)

*GDP* general dental practitioner, *Resto*. restorative specialist, *Endo*. Endodontist, *PSP* post space preparation, *ETT* endodontically treated teeth; Rarely: 0–20%; Sometimes: 30–50%; More frequently: 60–100%

### Agreement between knowledge and practice

The agreement levels between the knowledge and practice responses of the participants were statistically significant in all aspects (*p* < 0.05) (Table 4). Of the ten treatment aspects comparing each participant's knowledge and practice responses, none demonstrated "almost perfect" agreement. In contrast, only one (aspect #7, the time of PSP after obturation in the presence of a periapical lesion (PA) demonstrated a "strong" level of agreement. The remaining aspects showed either weak or minimal agreement levels (*p* < 0.0.53) (aspects #1, 3, 5, 6, and 9) or no agreement between knowledge and practice (#2, 4, 8, and 10) (Table 4).

### Differences between practitioners in the technical management of ETT

Table 2 shows the different participants' responses towards the technical management of restoring ETT; statistically significant differences were observed. A high proportion of endodontists (93.3%) believed that the clinician who performed the RCT is the best clinician to prepare the post space, as did 64.6% of restorative specialists and 42.5% of GDPs (*p* = 0.001).

**Table 2:** Association between the responses to the knowledge and practice aspects about the management of ETT requires post-placement with the specialty of a sample of Saudi dentists (n = 203)

Aspects	Knowledge n (%)			Χ^2^-value	*p* value	Practice n (%)			Χ^2^-value	*p* value
Technical management of ETT	GDP	Resto	Endo			GDP	Resto	Endo		
1. The best clinician to prepare the PS?				19.49	0.001*				4.99	0.287
The clinician who performed the root canal treatment	51(42.5)	44(64.7)	14(93.3)			26(21.7)	18(26.5)	1(6.7)		
The clinician who will fabricate the post	52(43.3)	16(23.5)	1(6.7)			75(62.5)	40(58.8)	9(60.0)		
Anyone; they are both equally qualified	17(14.2)	8(11.8)	0(0)			19(15.8)	10(14.7)	5(33.3		
2. Routine placement of a rubber dam during PSP?				1.97	0.374				27.62	< 0.001*
Yes	81(67.5)	42(61.8)	12(80.0)			24(20.0)	13(19.1)	12(80.0)		
No	39(32.5)	26(38.2)	3(20.0)			96(80.0)	55(80.9)	3(20.0)		
3. Recommended PSP technique?										
Rotary instruments (Gates Glidden, Peeso Reamers)	80(66.7)	42(61.8)	7(46.7)	35.91	< 0.001*	90(75.0)	59(86.8)	7(50.0)	37.3	< 0.001*
Post drills only	28(23.3)	6(8.8)	0			21(17.5)	6(8.8)	1(7.1)		
Heated endodontic instruments	2(1.7)	15(22.1)	4(26.7)			1(0.8)	2(2.9)	4(28.6)		
Other *(Chemical Solvents and others)*	10(8.4)	5(7.3)	4(26.7)			8(6.7)	1(1.5)	2(14.2)		
4. Minimum acceptable amount of gutta-percha remaining in the prepared canal?				2.98	0.562				0.92	0.922
≤ 3 mm	63(52.5)	33(48.5)	5(33.3)			13(10.8)	6(8.8)	1(6.7)		
4–5 mm	53(44.2)	33(48.5)	10(66.7)			98(81.7)	56(82.4)	12(80.0)		
More than 5 mm	4(3.3)	2(2.9)	0			9(7.5)	6(8.8)	2(13.3)		
5. Acceptable gap, if any, between the gutta-percha and the post?				3.85	0.427				3.67	0.453
No gap between the gutta-percha and the post	43(35.8)	33(48.5)	7(46.7)			47(39.2)	27(39.7)	7(46.7)		
A gap of 0–2 mm	71(59.2)	33(48.5)	8(53.3)			70(58.3)	36(52.9)	8(53.3)		
A gap of > 2 mm	6(5.0)	2(3.0)	0			3(2.5)	5(7.4)	0		
10. Placement of temporary crown over cemented indirect post and core for coronal seal				0.24	0.886				21.12	< 0.001*
Yes	89(74.2)	51(75.0)	12(80.0)			97(80.8)	62(91.2)	9(60)		
No	31(25.8)	17(25.0)	3(20.0)			23(19.2)	6(8.8)	6(40)		

*GDP* general dental practitioner, *Resto*. restorative specialist, *Endo*. endodontist, *PSP* post space preparation, *ETT* endodontically treated teeth

^*^Statistically significant at *p* < 0.05

Participants reported that a rubber dam should be routinely placed during PSP without any statistically significant difference across practitioner type (*p* = 0.374); however, in practice, endodontists put them routinely significantly more than the other practitioners (*p* < 0.001).

### Differences between practitioners in time-related aspects

Overall, most of the responses favored decreasing the time duration between the different treatment steps. However, there was a statistically significant difference in practitioners' responses from other disciplines concerning the time interval for restoring an ETT that requires post-placement (Table 3).

**Table 3:** Association between the responses to time-related knowledge and practice during the restoration of ETT and post-placement with the specialty of a sample of Saudi dentists (n = 203)

Aspects	Knowledge n (%)			Χ^2^-value	*p* value	Practice n (%)			Χ^2^-value	*p* value
Time-related aspects	GDP	Resto	Endo			GDP	Resto	Endo		
6. Time from obturation to start PSP?				5.96	0.427				28.69	< 0.001*
Immediately after obturation	29(24.2)	24(35.3)	7(46.7)			15(12.5)	6(8.8)	9(60.0)		
1–7 days after obturation	48(40.0)	26(38.2)	4(26.7)			74(61.7)	39(57.4)	5(33.3)		
2–4 weeks after obturation	20(16.7)	8(11.8)	1(6.7)			18(15.0)	12(17.6)	0(0)		
More than a month after obturation or duration of time is irrelevant	23(19.2)	10(14.7)	3(20.0)			13(10.8)	11(16.2)	(6.7)		
7. Time of PSP in presence of periapical lesion?				58.40	< 0.001*				50.68	< 0.001*
Immediately after RCT	7(5.8)	15(22.1)	10(66.7)			6(5.0)	15(22.1)	10(66.7)		
One week after RCT	9(7.5)	18(26.5)	2(13.3)			15(12.5)	16(23.5)	2(13.3)		
6 months after RCT	20(16.7)	7(10.3)	1(6.7)			19(15.8)	9(13.2)	1(6.7)		
Until there is evidence of periapical healing	84(70.0)	28(41.2)	2(13.3)			80(66.7)	28(41.2)	2(13.3)		
8. Acceptable time from PSP until cementing the cast post and core?				6.24	0.397				6.27	0.547
Less than 1 week	46(38.3)	31(45.0)	9(60.0)			25(21.9)	17(25.4)	0^†^		
1 week	42(35.0)	26(38.2)	4(26.7)			42(36.8)	32(47.8)	4(66.7)		
2 weeks	30(25.0)	9(13.2)	2(13.3)			28(24.6)	11(16.4)	1(16.7)		
More than 2 weeks	2(1.7)	2(2.9)	0			19(16.7)	7(10.4)	1(16.7)		
9. Acceptable time duration from post cementation until cementation of the final crown?				18.27	0.006*				17.39	0.008*
Less than 1 week	33(27.5)	12(17.6)	3(20.0)			22(19.1)	3(4.5)	0^†^		
From 1–2 weeks	61(50.8)	28(41.2)	6(40.0)			52(45.2)	28(41.8)	0		
From 2–4 weeks	25(20.8)	18(26.5)	5(33.3)			32(27.8)	23(33.8)	3(75.1)		
More than 1 month	1(0.8)	10(14.7)	1(6.7)			9(7.8)	13(19.1)	1(31.2)		

*GDP* general dental practitioner, *Resto*. restorative specialist, *Endo*. Endodontist, *PSP* post space preparation, *ETT* endodontically treated teeth

^*^Statistically significant at *p* < 0.05. ^†^Remaining endodontists did not answer since the situation/question did not apply to them, and they were excluded from the statistical test

Restorative specialists and endodontists preferred to prepare the post space immediately or shortly following obturation compared with GDPs. If a periapical lesion was present, endodontists significantly (*p* < 0.001) preferred immediate PSP compared with the other groups, who delayed it until there was evidence of healing.

### Effect of years of experience and workplace

The results showed no statistically significant differences in the relationship between the participants' responses (knowledge and practice) and years of experience or work sector for most of the above aspects. There was a statistically significant difference only in considering the routine application of a rubber dam during PSP. Nearly 71% of participants with less than five years of experience adopted this isolation concept compared with participants with more experience (6–10 years, 54.2% and more than ten years, 43.5%) (*X*^2^ = 7.87, *p* = 0.02).

## Discussion

### Agreement between knowledge and practice

This study measured the consistency in participants' responses between knowledge and practice aspects based on a presented case that required restorative management of ETT with an indirect post and core unit, followed by a crown. Nine out of ten aspects in the questionnaire showed "weak" agreement levels and below. The agreement was measured using intrarater reliability instead of comparing the total responses of the participants. Therefore, although the total numbers of the selected answers in practice and knowledge may be similar, there was disparity among the individual participants who selected these answers, resulting in minimal agreement (0.34), as observed in aspect #5 (Table 4). Conversely, in aspect #7, the participants' responses for both knowledge and practice were consistent, which resulted in strong agreement (0.804). This analysis provided valuable insight into the differences in each participant's attitudes towards a single clinical decision.

**Table 4:** Comparison of knowledge and practice answers and agreement levels among a sample of Saudi dentists (n = 203)

Aspects	Knowledge answers	Practice answers	Agreement* level
Technical management of ETT	n (%)	n (%)	
1. The best clinician to prepare the post space?			0.306
The clinician who performed the root canal treatment	109 (53.7)	45 (22.2)	Minimal
The clinician who will fabricate the post	69 (34.0)	124 (61.1)	*p* < 0.001
Anyone, they are both equally qualified	25 (12.3)	34 (16.7)	
2. Routine placement rubber dam during post space preparation?			0.192
Yes	135 (66.5)	49 (24.1)	None
No	68 (33.5)	154 (75.9)	*p* < 0.001
3. Recommended post space preparation technique?			0.521
Rotary instruments (Gates Glidden, Peeso Reamers)	129 (63.9)	156 (77.2)	Weak
Post drills only	34 (16.8)	28 (13.9)	*p* < 0.001
Heated endodontic instruments	21 (10.4)	7 (3.5)	
Other *(Chemical Solvents and others)*	18 (8.9)	11 (5.4)	
4. Minimum acceptable amount of gutta-percha remaining in the prepared canal?			0.176
≤ 3 mm	101 (49.8)	20 (9.9)	None
4–5 mm	96 (47.3)	166 (81.8)	*p* < 0.001
More than 5 mm	6 (3.0)	17 (8.4)	
5. Acceptable gap, if any, between the gutta-percha and the post?			0.344
No gap between the gutta-percha and the post	81 (39.9)	83 (40.9)	Minimal
A gap of 0 to 2 mm	114 (56.1)	112 (55.2)	*p* < 0.001
A gap of > 2 mm or presence of a gap does not matter	8 (3.9)	8 (3.9)	
*Time-related aspects*			
6. Time from obturation to start preparing the post space?			0.438
Immediately after obturation	30 (14.8)	60 (29.6%)	Weak
1–7 days after obturation	118 (58.1)	78 (38.4%)	*p* < 0.001
2–4 weeks after obturation	30 (14.8)	29 (14.3%)	
More than a month after obturation or duration of time is irrelevant	25 (12.3)	36 (17.7%)	
7. Time of PSP in presence of periapical lesion			0.804
Immediately after RCT	32 (15.8)	31 (15.3)	Strong
One week after RCT	29 (14.3)	33 (16.3)	*p* < 0.001
6 months after RCT	28 (13.8)	29 (14.3)	
Until there is evidence of periapical healing	114 (56.2)	110 (54.2)	
8. Acceptable time from PSP until cementing the indirect post and core?			0.128
Less than 1 week	79 (42.2)	42 (22.5)	None
1 week	66 (35.3)	78 (41.7)	*p* = 0.003
2 weeks	38(20.3)	40 (21.4)	
More than 2 weeks	4 (2.1)	27 (14.4)	
9. Acceptable duration from post cementation until cementation of the final crown?			0.301
Less than one week	44 (23.7)	25 (13.4)	Minimal
From 1–2 weeks	89 (47.8)	80 (43.0)	*p* < 0.001
From 2–4 weeks	42 (22.6)	58 (31.2)	
More than one month	11 (5.9)	23 (12.4)	
10. Placement of temporary crown over cemented post and core for coronal seal			0.156
Yes	152 (74.9)	165 (83.1)	None
No	51 (25.1)	38 (18.7)	*p* = 0.024

*PSP* post space preparation, *ETT*:endodontically treated teeth, *RCT* root canal treatment

^*^Agreement value measured by kappa statistics

There are few reports from healthcare literature on the discrepancy between what is known and what is done. For example, Khan et al. demonstrated that students may be knowledgeable of the shortened dental arch as a treatment concept but rarely implement it in clinical practice [20]. In obstetrics and gynecology, Wilder et al. found that although most obstetricians are aware of pregnancy and periodontal disease interrelationships, they rarely address such issues during patient care [21]. This difference has often been explained through knowledge translation, which hypothesizes that practice is a translation of knowledge, and knowledge is acquired first and then applied over time [22]. Therefore, factors that affect this process at any step may contribute to the gap. Adams et al. estimated this gap in following medical guidelines to be approximately 27%. Several researchers have explored the gap and its related elements [23]. In their informative and comprehensive review, Afrashtehfar and Assery discussed the challenges a dentist faces in practicing evidence-based dentistry. Some of the issues are related to restrictions in accessing relevant information and its critical assessment and the absence of an applicable model to permit a clinical shift and subsequent integration, and finally, patient hindrance limitations [16]. Majumdar et al. categorized the barriers to applying evidence to clinical care into four main categories: evidence, clinician, patient and setting [15]. To demonstrate this, participants' answers to aspect #7, which was related to the duration of time between obturation and PSP in the presence of a PA, demonstrates a barrier in "evidence", despite the strong agreement (0.804). In cases with a PA lesion, most participants believed and opted for delaying PSP until demonstration of PA healing, which does not follow current recommendations of restoring ETT as soon as possible [24]. This issue with their information or "evidence" was reflected in their practice as well. Examples of barriers during application include information aspect #2. Most of the participants answered that rubber dam isolation should be applied during PSP as recommended in the literature [25]; however, participants rarely applied this principle. Although being aware of new evidence is a prerequisite to changing practice, gaps or inconsistencies between best practice and daily practice are not entirely a result of knowledge deficits or vice versa. Some participants demonstrated sound clinical practice, while their answers to the related knowledge questions were erroneous. In responding to aspect #4 regarding the acceptable remaining amount of gutta-percha apically during PSP, half of the participants thought that less than 3 mm was acceptable, while in practice, only 10% did so, while the remaining 80% of the participants left 4–5 mm of root filling material after PSP in clinical practice, as recommended in the literature [1, 26], which does not follow the knowledge translation model. Additionally, in some respects, several options are considered clinically acceptable. Yet, participants' choices were inconsistent in their responses between the knowledge and practice aspects, such as the PSP technique or the time from obturation to start of PSP (aspects #3 and #6 in Table 4). Though it was not within this study's scope, it is essential to understand why participants shifted their responses.

Furthermore, this lack of compliance existed despite 72% of the participants being recent graduates with less than 5 years of experience; one would assume that such new clinicians are likely equipped with the latest evidence. The vital role of education, the impact of curricula, and the role of continuous professional development cannot be overemphasized. However, neither experience nor age showed statistically significant differences in this study, implying that other factors affect the results.

In addition to knowledge, Cabana et al. highlight the role of attitudes and behaviors as barriers to the application, too [27]. The Theory of Planned Behavior has been investigated as a model to explain health care professionals' intention, from different specialties, to apply the same clinical guidelines into practice [28]. Bonetti et al. explored the behavior of dentists. They found that together, intentions and habits, as applied by both the Theory of Planned Behavior and Operant Learning theory, can explain the majority of variance in dentists' clinical behavior [29]. However, further investigations are needed to understand better why dentists practice differently from what they know since each setting is independent.

### Differences between practitioners

Research has repeatedly demonstrated different approaches among different disciplines and between specialists and GDPs [30, 31]. This study focused on the overlapping area between RCT and prosthodontic treatment during PSP, which can be performed by any of the practitioners from the disciplines investigated. The results demonstrated significant differences among the disciplines in several aspects of this study.

For example, specialists favored more conservative and safer options, as demonstrated by the PSP technique's choice. Although all groups favored rotary instruments, the most conservative option was heated instruments, mostly used by endodontists (26.8%). Interestingly, many studies have indicated no difference in leakage when removing the gutta-percha with a heated or rotary instrument [1, 32, 33].

The differences in approach between the different practitioners may become crucial if the approaches are against EBD, e.g., many participants thought it best to wait for evidence of periapical healing before initiating PSP in the presence of a PA lesion. The difference was highly statistically significant between the groups. Most endodontists believed that immediate PSP was best, while most GDPs thought it best to delay treatment until there was evidence of healing. The responses of restorative specialists were equally divided between these two options. Outcome studies of endodontic treatment have determined that an adequate coronal seal is as important as the quality of RCT for success. PAs take several months to many years to heal [34, 35]; hence, delaying cuspal coverage, even for a few months, results in significantly more ETT failures and fractures [24, 36]. Clinical studies have also demonstrated that the presence of a post did not affect the outcome of ETT in the presence or absence of a PA lesion [2, 37–39]. Additionally, Yee et al. demonstrated that the highest survival in ETT was when the post and core unit were applied within two months after primary RCT, and the crown was placed within 2 months after that [36]. Therefore, placement of a post whenever indicated should not be delayed.

Furthermore, 60% of endodontists immediately prepared the post space after obturation, while GDPs and restorative specialists delayed PSP from a few days to more than a month. PSP at the time of RCT presents the advantage of easier removal of the root canal filling before it sets, benefiting from working length determination and familiarity with the canal's anatomical characteristics and the already-present rubber dam isolation and minimizing leakage [1, 24, 40].

Following the indirect post and core cementation, 74.9% of the study participants thought that the cemented indirect post and core unit alone did not provide an adequate coronal seal. In practice, significantly more restorative specialists (91.2%) placed temporary crowns than GDPs (80.8%). Although temporary crowns fulfil other purposes than providing a coronal seal, such as esthetic, functional, and occlusal purposes and proximal stability, prolonged placement of temporary crowns might result in the cement's microleakage jeopardizing the success of RCT. Several studies have shown that the root canal system can become rapidly reinfected in the absence of a satisfactory coronal seal [41, 42].

Overall*,* for most of the time-related aspects, participants revealed a positive trend following recommended evidence that higher success is achieved when placing a definitive restoration within the minimum treatment duration to minimize the possibility of coronal leakage.

Advances in digital dentistry have promoted restorative treatment in terms of time efficiency and quality [43]. The use of digital impressions and CAD/CAM fabrication can help the restorative dentist seal the filled root canal system and protect the tooth as soon as possible. A customized post, if indicated, could be designed, milled, and cemented with minimal laboratory steps. Then, a temporary or definitive restoration is fabricated chairside and cemented in the same visit.

This study measured the agreement between dental practitioners' knowledge and practice, which the authors have not found to be addressed in the literature. There were generally poor agreement levels between dentists' knowledge and practice in aspects related to PSP when restoring ETT. These findings highlight that studies investigating participants' knowledge on any issue do not necessarily reflect what the participants may practice, and practice does not reflect knowledge. Further research is needed to identify the reasons behind this disagreement among dental practitioners and address any possible barriers to minimize a care gap.

Additionally, to reduce variation and control the participants' responses, this study's questions were relevant to only one standard clinical scenario that could be treated in any clinical setting. Setting a different clinical scenario with advanced or controversial treatment options may generate different responses from the participants.

The participants' specialty affected both their knowledge and their clinical practice; however, the disagreements between knowledge and practice were demonstrated among all groups in this sample. This finding highlights the importance of a multidisciplinary approach in managing cases and periodically updating dentists in aspects of other specialties that directly affect their practice, which might improve the success rate of dental treatment [44]. Unfortunately, further analysis of agreement among the different practitioners was not possible due to the small number of participating endodontists. Although, the number of each subgroup could not be retrieved accurately from the SDS archives, the distribution of the different specialties within our sample was similar to that of the Saudi dental population [45]. The numbers of participating clinicians practising in private clinics, and those above the age of 40, were also low and need to be addressed in the future. The lower age might represent the age of the members in the society, or the enthusiasm of younger professionals to participate. Nevertheless, the correlation of the participants' age and their responses was investigated, and no statistical significance was found. Finally, it is also important to acknowledge that the results are as accurate as the participants' responses and may be influenced by a bias response bias.

## Conclusion

Within the limits of our study and based on the participants' responses to questions on a selected clinical scenario involving an indirect post-placement in posterior ETT:

1. Only one out of ten aspects showed strong agreement between knowledge and practice methods among individual participants.
2. The participants' specialty influenced their responses to both knowledge and clinical practice.

## Supplementary Information

**Additional file 1**. Sample of the questionnaire distributed to a number of Saudi dentists

## Data Availability

All data analyzed during this study are included in this published article. Additionally, the complete dataset supporting the conclusions of this article is available from the corresponding author and can be accessed upon a reasonable request.

## Abbreviations

RCT: Root canal treatment
PSP: Post space preparation
ETT: Endodontically treated teeth
GDP: General dental practitioner
PA: Periapical
EBD: Evidence-based dentistry
