# Supplementary material for: Do dentists practice what they know? A cross-sectional study on the agreement between dentists' knowledge and practice in restoring endodontically treated teeth
Source: BMC Oral Health. 2021 Mar 10;21:110. doi: 10.1186/s12903-021-01479-2 (PMC7945671; doi:10.1186/s12903-021-01479-2)
Supplement: Supplementary file 1 — Additional file 1. Sample of the questionnaire distributed to a number of Saudi dentists [file 12903_2021_1479_MOESM1_ESM.docx]

**Part 1: Personal Data**

1. Age:……….
2. Gender:
3. Male
4. Female
5. Nationality:
6. Saudi
7. Non-Saudi. (Please specify):… …..
8. What is your specialty?
9. General Practitioner.
10. Prosthodontist
11. Endodontist
12. Restorative specialist/ SBARD/AEGD
13. Other (Please specify):…….
14. How long have you been practicing your specialty?………..
15. Where do you primarily work?
    1. Governmental institiution.
    2. Academic institution.
    3. Private practice.

**Part 2:**

1. How frequently do you crown (or refer for crowning) endodontically treated posterior teeth?

| 0% | 10% | 20% | 30% | 40% | 50% | 60% | 70% | 80% | 90% | 100% |
| --- | --- | --- | --- | --- | --- | --- | --- | --- | --- | --- |

1. Do you think all endodontically treated posterior teeth require cuspal coverage restoations?

| 0% | 10% | 20% | 30% | 40% | 50% | 60% | 70% | 80% | 90% | 100% |
| --- | --- | --- | --- | --- | --- | --- | --- | --- | --- | --- |

1. How often do you place a post in posterior endodontically treated teeth?

| 0% | 10% | 20% | 30% | 40% | 50% | 60% | 70% | 80% | 90% | 100% |
| --- | --- | --- | --- | --- | --- | --- | --- | --- | --- | --- |

**Part 3 The following questions are related to treatment of an endodontically treated tooth which is indicated for an indirect post and core and crown, and does not require crown lengthening. Please answer the following questions accordingly:**

1. When do *you* mostly start preparing the post space?
2. Immediately after obturation.
3. 1-7 days after RCT.
4. 2-4 weeks after RCT.
5. More than a month after RCT.
6. When do you think it is best to start preparing the post space?
7. Immediately after obturation.
8. 1-7 days after RCT.
9. 2-4 weeks after RCT.
10. More than a month after RCT.
11. Duration of time is irrelevant.
12. If a periradicular lesion is present in the case above, when would you prepare the post space?
    1. Immediately after RCT.
    2. One week after RCT.
    3. 6 months after RCT.
    4. Until there is evidence of periapical healing.
13. If there is a periapical lesion in an endodontically treated tooth, when do you think the post space should be prepared?
    1. Immediately after RCT.
    2. One week after RCT.
    3. 6 months after RCT.
    4. Until there is evidence of periapical healing.
14. In your practice, who mostly does the post space preparation?
    1. The clinician who performed the root canal treatment.
    2. The clinician who will fabricate the post.
    3. Anyone, they are both equally qualified.
15. Who do you think is the best clinician to prepare the post space?
    1. The clinician who performed the root canal treatment.
    2. The clinician who will fabricate the post.
    3. Anyone, they are both equally qualified.
16. Do you routinely apply rubber dam during post space preparation?
    1. Yes.
    2. No.
17. Do you think it is recommended to routinely place rubber dam during post space preparation?
    1. Yes.
    2. No.
18. What is the main technique that *you* use in preparing the post space?
    1. Rotary instruments (Gates Glidden, Peeso Reamers).
    2. Post drills only.
    3. Heated endodontic instruments.
    4. Chemical solvents.
    5. Other (Please specify):….
19. What do you think is the recommended technique for preparing the post space?
    1. Rotary instruments (Gates Glidden, Peeso Reamers).
    2. Post drills only.
    3. Heated endodontic instruments.
    4. Chemical solvents.
    5. Other (Please specify):….
20. After post space preparation, what is the remaning amount of gutta-percha that you would leave in a canal?
    1. 3mm or less.
    2. 4-5mm.
    3. More than 5mm.
21. What do you think the minimum acceptable amount of gutta-percha remaining in the prepared canal?
    1. 3mm or less.
    2. 4-5mm.
    3. More than 5mm.
22. How much of a gap between the gutta percha and the post do you accept?
    1. No gap between the gutta percha and the post.
    2. A gap of 0 to 2mm.
    3. A gap of > 2mm.

1. What do you think is the acceptable gap, if any, between the gutta percha and the post?
   1. No gap between the gutta percha and the post.
   2. A gap of 0 to 2mm.
   3. A gap of > 2mm.
2. How long do your cases usually take from post space preparation until cementation of the indirect post and core?
   1. Less than one week.
   2. One week.
   3. 2 weeks.
   4. More than 2 weeks.
   5. Not applicable.
3. How long do you think it is acceptable to take from post space preparation until cementation of the indirect post and core?
   1. Less than one week.
   2. One week.
   3. 2 weeks.
   4. More than 2 weeks.
4. How long does it mostly take your cases from cementation of the post untill cementation of the final crown?
   1. Less than one week.
   2. From 1-2 weeks.
   3. From 2-4 weeks.
   4. More than 1 month.
   5. Not applicable.
5. What do you think is the acceptable time duration from post cementation until cementation of the final crown?
   1. Less than one week.
   2. From 1-2 weeks.
   3. From 2-4 weeks.
   4. More than 1 month.
6. Do you place (or request) a temporary crown after cementing an indirect post and core, to provide a coronal seal?
   1. Yes.
   2. No.
7. Do you think a temporary crown is needed to coronaly seal a cemented indirect post and core?
   1. Yes.
   2. No.

Thank you
